# Eosinophilic Solid and Cystic Renal Cell Carcinoma: From Unclassified to Classified, A Case Report

**DOI:** 10.5146/tjpath.2021.01531

**Published:** 2022-01-21

**Authors:** Rashim Sharma, Balamurugan Thirunavukkarasu, Poonam Elhence, Mahaveer Singh Rodha, Binit Sureka

**Affiliations:** Departments of Pathology and Lab Medicine, All India Institute of Medical Sciences, Rajasthan, India; Departments of Trauma and Emergency, All India Institute of Medical Sciences, Rajasthan, India; Departments of Diagnostic and Interventional Radiology, All India Institute of Medical Sciences, Rajasthan, India

**Keywords:** Eosinophilic solid and cystic renal cell carcinoma, Unclassified renal cell carcinoma, Emerging entity, Oncocytic renal tumour

## Abstract

Eosinophilic solid and cystic renal cell carcinoma (ESC RCC) is a novel tumour with unique morphological and immunohistochemical features. It is a recently described entity after the 2016 World Health Organization Classification of Tumours of the Urinary System and Male Genital Organs and is characterised by a solid cystic tumour composed of polygonal cells with voluminous eosinophilic cytoplasm and CK20 positivity. This tumour has uncertain malignant potential and also has an association with tuberous sclerosis complex (TSC). Sarcomatoid differentiation has not been reported in ESC RCC till now. ESC RCC poses a diagnostic challenge as many eosinophilic/oncocytic renal tumours are included in the differentials. We present a case of ESC RCC with sarcomatoid differentiation in an elderly female without any clinical features of TSC and discuss the differential diagnosis of oncocytic renal tumours.

## INTRODUCTION

Our understanding of renal tumours has significantly improved in the past years. This is due to advancements in the field of molecular pathology. Several new entities have been described and there is reclassification of the existing tumours after consideration of clinical features, morphology, immunohistochemistry and genetic alterations. Oncocytic renal tumours, that were once in the unclassified category, have gained a separate diagnostic category owing to their prognostic implication and clinical relevance. Attention to the histomorphology and a methodical immunohistochemical approach can lead to an accurate diagnosis in many such tumours.

## CASE REPORT

**Figure 1:**
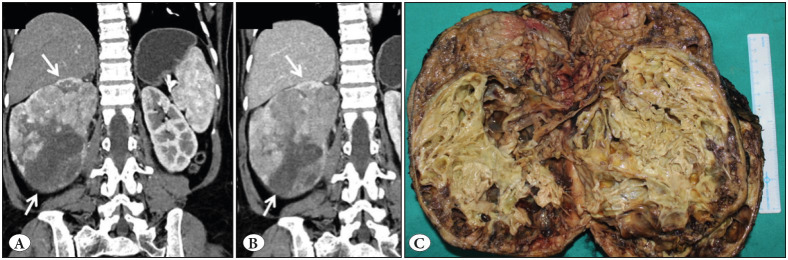
**A)** Coronal CT image in corticomedullary phase shows large malignant mass in the right kidney (arrows) with heterogeneous moderate enhancement and a large area of necrosis. **B)** Nephrographic phase scan showing same pattern of moderate enhancement with no washout (arrows). **C)** Growth is solid cystic, predominantly yellowish and necrotic in the inferior and middle poles and fleshy tan brown in the upper pole.

A 67-year-old female presented with a gradually increasing abdominal lump for 7 months. On clinical examination, a palpable lump was noted in the right lumbar region. Urine testing for malignant cytology was negative on three consecutive samples. Computed Tomography urography revealed a large, heterogeneously enhancing mass measuring 18x16x15 cm in the inferior pole of right kidney and causing superior displacement of the remaining kidney with splaying of the pelvicalyceal system** **(Figure 1A).** **There was heterogeneous moderate enhancement in the corticomedullary phase and the same pattern of moderate enhancement in the nephrographic phase with no washout in the delayed phase (Figure 1B). A large central non-enhancing region was also noted suggesting necrosis along with multiple enhancing septations. The lesion was displacing the infrahepatic inferior vena cava, pancreas and second part of duodenum to the left side with no evidence of metastasis. The patient subsequently underwent radical nephrectomy.

**Figure 2:**
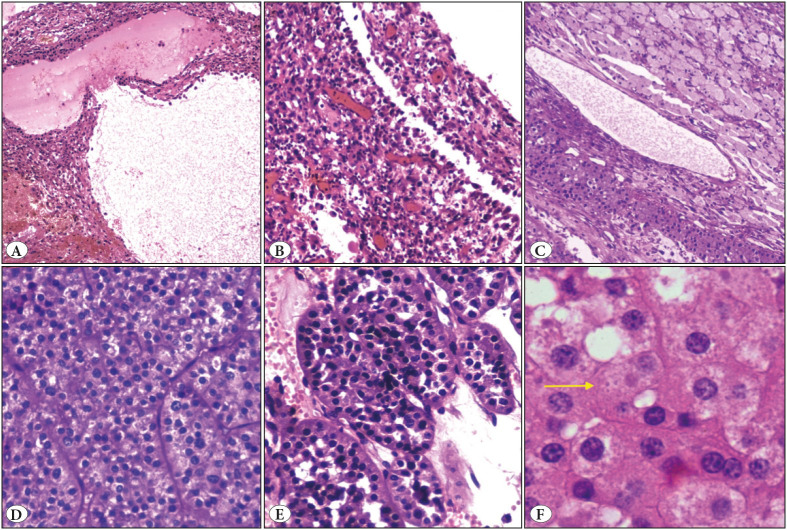
**A)** Cystic area (H&E; x4). **B)** Polygonal tumour cells with hobnailing (H&E; x20). **C)** Tumour cells admixed with foamy macrophages and scattered lymphocytes (H&E; x20). **D)** Solid arrangement of tumour cells with vacuolated eosinophilic cytoplasm (H&E; x40). **E)** Nested/archipelagenous pattern with dense cytoplasm (H&E; x40). **F)** Intracytoplasmic amphophilic leishmania-like inclusions in the tumour cells (yellow arrow) (H&E; x60)

**Figure 3:**
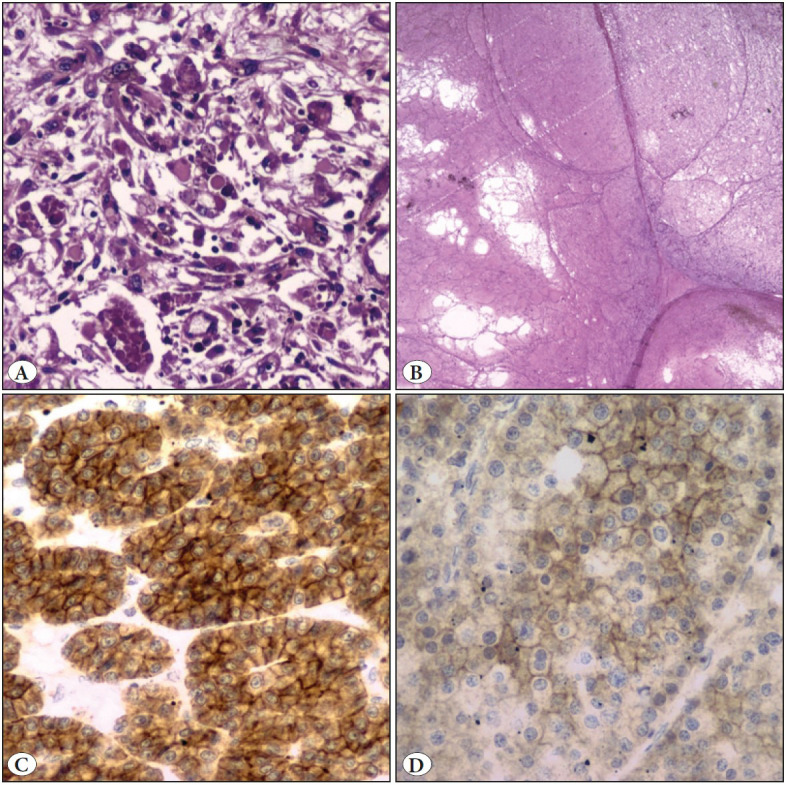
**A)** Sarcomatoid areas noted in the tumour (H&E; x40). **B)** Large area of tumour necrosis (H&E; x20). **C)** Strong and diffuse membranous CD117 positivity in the nested area (IHC; x40). **D)** Weak membranous CD117 positivity in the solid area (IHC; x40).

On gross examination, the kidney measured 21x16x12cm. The attached adrenal gland was unremarkable. On serial sectioning, an ill-circumscribed solid cystic tan brown, yellowish tumour was noted in the middle and inferior pole, abutting the capsule and measuring 19.5x14x9.5 cm (Figure 1C). Two coalescing nodules were noted. The larger nodule was predominantly yellowish cystic whereas the smaller nodule showed solid-grey brown area. The cystic spaces ranged from 0.3 to 1.6 cm. The renal sinus was pushed to upper pole and appeared free. Areas of necrosis were noted. On microscopy, the macrocysts were lined by round to polygonal tumour cells with eosinophilic granular cytoplasm, round nuclei, coarse to hyperchromatic nuclei and conspicuous nucleoli (Figure 2A,B). Lymphocytes and foamy histiocytes were interspersed with the tumour cells (Figure 2C). The solid area showed tumour arranged in nests/insular/archipelagenous, trabecular and solid pattern (Figure 2D,E). Cells with voluminous cytoplasm, nuclear pleomorphism and hobnailing were noted predominantly in the cystic areas, while the cytoplasm was dense, bright eosinophilic in the organoid areas. Focally, intracytoplasmic amphophilic to basophilic leishmania-like inclusions with halo were also seen (Figure 2F). However, papillary pattern, abundant clear cytoplasm, perinuclear halo, psammoma bodies, raisinoid nuclei, and biphasic cellular population were absent. Sarcomatoid differentiation in the form of spindling (10%) and areas of necrosis (40%) were seen (Figure 3A,B). The renal sinus, pelvis, adrenal gland and renal vessels were free of tumour.

**Figure 4:**
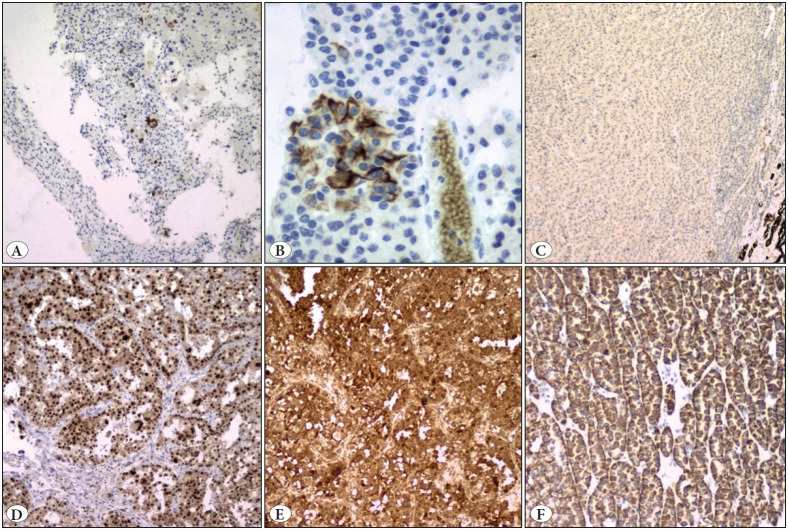
**A,B)** CK20 focal strong membranous positivity in cystic area (IHC; x10 and x40). **C)** CK7 is negative in the tumour area (positive internal control - distal convoluted tubules) (IHC; x10). **D)** PAX-8 positivity (IHC; x20). **E)** Melan-A positivity (IHC; x20). **F)** Pancytokeratin positivity (IHC; x20).

The differentials considered were oncocytic renal tumours such as eosinophilic solid and cystic renal cell carcinoma, eosinophilic variant of chromophobe renal cell carcinoma, hybrid oncocytic/chromophobe tumour (HOCT), low grade oncocytic tumour (LOT), high grade oncocytic tumour (HOT), ALK rearranged renal cell carcinoma, MiT family translocation renal cell carcinoma, and epithelioid angiomyolipoma (AML). Immunohistochemistry (IHC) was done (Figure 4). The tumour cells were diffusely and strongly positive for PAX8 (MRQ-50, Cell Marque, RTU), Pan cytokeratin (AE1/AE3, Thermofisher, RTU). Tumour cells showed focal strong positivity for CK20 (Ks20.8, Thermofisher, RTU) predominantly in the cystic areas. There was variable expression of CD117 (A4502, Dako, 1:200 dilution) in the nested areas (intense) vs. solid area (weak positive) (Figure 3C,D). Tumour cells were also positive for CD10 (GM003, PathnSitu, RTU), and Melan A (A103, Thermofisher, RTU). They were negative for CK7 (OV-TL12/30, Thermofisher, RTU), AMACR (13H4, Thermofisher, RTU), S100P (4C4.9, Thermofisher, RTU), TFE3 (MRQ-37, Cell Marque, RTU), and ALK (CD246, Dako, RTU). Based on the histomorphology and IHC, a diagnosis of eosinophilic solid and cystic renal cell carcinoma, pT2bN0 was rendered. There were no post-operative complications and currently the patient is on routine post-operative care.

## DISCUSSION

The term “Eosinophilic solid and cystic renal cell carcinoma (ESC RCC)” was coined by Trpkov et al in 2016 (1). Once described under unclassified RCC/eosinophilic/oncocytic tumours, the characteristic features of ESC RCC were later described in a series illustrating the various RCC in patients with tuberous sclerosis (TSC associated RCC) (2,3). Subsequently, tumours with identical morphology were also described in sporadic cases, exclusively in females (1). As the name states, ESC RCC are characterised by solid and cystic areas. Both micro and macrocysts lined by tumour cells with or without hobnailing can be seen. Though predominantly described in adult females, cases have been described in teenagers and in males (1,4). Grossly, size of most of the tumours are less than 5 cm (Range: 0.5 cm to 13.5 cm). Size of tumour in the present case was 19.5 cm which is the largest documented till date** **(1,2). The classically described features such as nested and solid architecture, hobnailing, voluminous granular cytoplasm with amphophilic to basophilic “leishmania-like” inclusions were also identified in the present case (1,2,5). The signature IHC profile in ESC RCC is CK20 positivity (patchy or diffuse) with CK7 absent to weak positivity. CK20 can be negative in up to 12% of cases but it should never be diffuse CK7 positive with CK20 negative in ESC RCC. Our case showed strong CK 20 positivity, albeit focal. CD117 is usually negative. Focal positivity was described in 1 out of 16 patients in a series (1). In the present case, CD117 expression was limited to nested “Oncocytoma-like region” and weak to absent in the solid areas.

ESC RCC has a wide range of differentials to be considered. The eosinophilic renal neoplasms include oncocytoma, eosinophilic variant of chromophobe RCC, hybrid oncocytic/chromophobe tumour (HOCT), MiT family translocation carcinoma, clear cell RCC, epithelioid angiomyolipoma, low grade oncocytic tumour (LOT) and high grade oncocytic tumour (HOT)/ eosinophilic vacuolated tumour (EVT), Succinate dehydrogenase (SDH) deficient RCC, ALK rearranged RCC, and epithelioid angiomyolipoma. Other tumours less relevant to this case include tubulocystic RCC, acquired cystic disease associated RCC, Fumarate hydratase deficient RCC, papillary RCC, and thyroid-like follicular carcinoma of the kidney (5–7). Oncocytoma is a benign tumour characterised by monomorphic population of tumour cells. Though there were focal compact areas resembling oncocytoma in this case, there were other areas with variable architectural pattern with voluminous eosinophilic granular cytoplasm and cystic areas. This case lacked the perinuclear halo and the irregular hyperchromatic raisinoid nuclei of chromophobe renal cell carcinoma. CK7 was negative giving credence to exclusion of both these differentials and HOCT. ESC RCC can express Melan A, HMB45 or Cathepsin K. PAX-8 positivity rules out an epithelioid angiomyolipoma, in addition to absence of other morphological features of AML (2,6,8). There was absence of the characteristic “intracytoplasmic vacuoles/ flocculent cytoplasm” seen in SDH deficient RCC. In addition, diffuse pan cytokeratin positivity noted in this case negates the diagnosis. MiT family translocation carcinoma presents in a younger age group and can either be Xp11.2 or t (6,11) type. Few features shared by these tumours and ESC RCC are the polygonal cells with abundant vacuolated cytoplasm and positivity for Melan A. However, the macrocystic areas, lack of papillary pattern, CK20 positivity and negative TFE3 favours ESC RCC. ALK rearranged RCC shows variable architectural pattern, and mucinous myxoid stroma with or without signet ring cells with ALK positivity.

Other recently described oncocytic tumours are LOT and HOT/EVT. LOT is grossly predominantly solid, and tan yellow. Microscopy resembles that of oncocytoma. However, they are negative for CD117 and are diffusely positive for CK7 (7). High-grade oncocytic tumour was recently relabelled as “Eosinophilic vacuolated tumour” characterised by large vacuolated cytoplasm and higher nuclear grade with positivity of pan CK, PAX8 and CD117 (9). CK7 is negative to focal weak positive. The cells lack the granular cytoplasm of ESC RCC and are negative for Melan A and CK20 (9).

Tubulocystic RCCs are dominantly microcystic along with tightly cohesive tubules lined by eosinophilic cells. Hobnailing can also be seen. They are diffusely positive for CK7, AMACR, CD10, EMA, Vimentin and PAX8 (10). Fumarate hydratase deficient RCC characteristically has papillary pattern and prominent nucleoli with perinuclear halo. Acquired cystic kidney disease associated RCC is seen mainly in end stage renal disease (11). Morphologically variable patterns are noted with solid, microcystic and macrocystic areas. Cells have prominent nucleoli along with numerous oxalate crystals. On IHC, these tumours are positive for CD10, AMACR and negative for CK7 (6).

In conclusion, eosinophilic solid and cystic renal cell carcinoma (ESC RCC) is one of the tumours described post 2016 WHO classification and is classified as a “novel entity” by the genitourinary pathology society (GUPS) (9,12). So far, approximately 65 cases have been reported in several case series (1–4,8,13–17). This is probably an understatement as many oncocytic tumours in the archives are being reviewed and recognised. Currently, WHO nuclear grading is not recommended. The exact prognosis and metastatic potential are yet to be determined as few reports have documented metastases in ESC (4,8,17). Sarcomatoid differentiation has not been described in literature.

## Conflict of Interest

The authors declare no conflict of interest.

## Funding

None
